# Exploring the relationship between mothers' knowledge, attitudes, and complementary feeding practices and nutritional status of children aged 6–23 months in Jeddah, Saudi Arabia

**DOI:** 10.3389/fped.2026.1884189

**Published:** 2026-09-24

**Authors:** Wejdan M. Alfezzy, Buthaina M. Aljehany

**Affiliations:** Food and Nutrition Department, Human Sciences and Design Faculty, King Abdulaziz University, Jeddah, Saudi Arabia

**Keywords:** child nutritional status, complementary feeding, knowledge-attitudes-practices (KAP), mothers, Saudi Arabia

## Abstract

**Background:**

Proper complementary feeding (CF) during the first 1,000 days of life is essential for optimal growth and development. These CF practices are influenced by maternal knowledge, attitudes, and practices (KAP). The relationship between these parameters is not well investigated within the Saudi context.

**Objectives:**

To assess maternal KAP regarding CF and examine their association with the nutritional status of children aged 6–23 months in Jeddah, Saudi Arabia.

**Methods:**

A cross-sectional study was conducted in four well-baby clinics, including mothers with children aged 6–23 months. Data were collected using the Food and Agriculture Organization (FAO) for assessing nutrition-related Knowledge, Attitudes, and Practices (KAP). Children's anthropometric measurements (weight, length) were taken and converted to Weight-for-Age (WFA), Length-for-Age (LFA), and Weight-for-Length (WFL) Z-scores.

**Results:**

Of the 390 mothers participating in the study, 74.4% relied on digital media for information about CF. While 82.3% of children met minimum dietary diversity, only about 50% met minimum meal frequency recommendations. According to WFL, 6.7% of children were overweight. WFA was significantly correlated with maternal nutritional knowledge (r = 0.18, *p* < 0.001), attitude (r = 0.11, *p* = 0.031), and complementary feeding practices (r = 0.11, *p* = 0.028). In multivariable analyses, maternal BMI (*β* = 0.016, *p* < 0.001) and nutritional knowledge (*β* = 0.041, *p* = 0.013) were positively associated with WFA. For LFA, maternal working status (vs. not working) had a negative association (*β* = –0.058, *p* = 0.046). In contrast, nutritional knowledge (*β* = 0.026, *p* = 0.048) showed a positive association. No significant associations were observed for WFL.

**Conclusion:**

Maternal nutritional knowledge was associated with weight-for-age and length-for-age, while maternal BMI was independently associated with weight-for-age. The small effect sizes suggest that these factors alone are unlikely to explain substantial variation in child growth. These findings support multifactorial approaches to complementary-feeding education and child-growth monitoring.

## 1 Introduction

Proper infant nutrition is vital for health from birth through adulthood. Nutrition in the first three years is particularly important, as it helps reduce illness and mortality, lowers the risk of chronic diseases, and supports healthy physical and mental development (1). Children aged 6 to 23 months experience a key stage of growth and development (2). Poor nutrition during this time can delay developmental milestones and increase the risk of long-term physical, cognitive, and health issues (3). The first 1,000 days of life, which include pregnancy and a child's second birthday, are recognized as a critical period for lifelong health. Nutrition during this time influences the risks of obesity, stunting, wasting, underweight, micronutrient deficiencies, and other health outcomes (4, 5). Important factors include maternal nutrition, breastfeeding, formula feeding, and complementary feeding (CF) (6). By around six months, breast milk alone does not sufficiently meet infants' nutritional needs, making the timely introduction of safe, varied, and adequate complementary foods essential (7, 8).

Complementary feeding means providing foods and liquids in addition to breast milk (9). Effective CF should be timely, nutritionally adequate, safe, and diverse, incorporating at least four food groups (10). However, global data show that fewer than one in four infants aged 6 to 23 months meet minimum standards for dietary variety and feeding frequency (7). Poor CF practices, such as introducing foods too early, limiting dietary variety, or feeding too infrequently, are a significant cause of malnutrition, illness, and child mortality worldwide (11). To keep track of progress in infant and young child feeding, the WHO established indicators in 2008 and updated them in 2021 to include measures of dietary diversity, food groups, and unhealthy food consumption (12). Since mothers are the main caregivers, their knowledge, attitudes, and practices (KAP) are crucial in shaping children's feeding patterns and nutritional outcomes (13, 14). Indeed, lack of maternal knowledge about what, when, and how to feed children remains a key driver of malnutrition (15).

In Saudi Arabia, national data show that among children under five, 6.1% were overweight, 11.8% were wasted, and 9.3% were stunted (16). Another study found that nearly 42% of children aged 6 to 23 months did not get the minimum number of meals daily, while 65% were not fed often enough or offered a diverse diet (17). In Unaizah, Saudi Arabia, research showed that while mothers had limited knowledge about CF, their practices were relatively adequate (18). Still, evidence is limited on how maternal KAP regarding CF relates directly to child nutritional status in Saudi Arabia. Improving maternal nutritional knowledge has consistently been linked to better child feeding practices and outcomes (19). Additionally, enhancing maternal KAP has been shown to reduce malnutrition and support healthy child growth (20). Global initiatives, like the United Nations' Second Report on the World Nutrition Situation, highlight adequate complementary feeding as a key part of child health strategies (16). In this context, and in line with Saudi Vision 2030—particularly the Health Sector Transformation Program, which focuses on improving health and quality of life (21)—this study aims to provide evidence on maternal KAP of CF and its relation with the nutritional status of children aged 6 to 23 months in Jeddah.

## 2 Methods

### 2.1 Study design and subjects

A cross-sectional study was conducted from January to July 2024 in well-baby clinics, at 4 health centers in Jeddah City, Saudi Arabia. The inclusion criteria consisted of all mothers with healthy children aged between 6 and 23 months. In addition, the children in this age range should be in good health at birth and regularly visit health centers for vaccinations and growth monitoring. The exclusion criteria included preterm babies, children with congenital malformations such as cleft palate or limb deformities, and children with chronic diseases or food allergies. These conditions can negatively affect CF practices and the nutritional status of the children. For mothers who had more than one child within the eligible age range (6–23 months), only one child per mother was included in the study, selected as the youngest eligible child, to avoid intra-family clustering of observations and preserve the statistical independence of observations assumed in the regression analyses (22).

This study was conducted in accordance with the Declaration of Helsinki. All research methods were carried out in accordance with the relevant guidelines and regulations. The study protocol was approved by the Biomedical Ethics Research Committee at King Abdulaziz University, Saudi Arabia (Reference No.428-23) and by the General Directorate of Research & Studies/Ministry of Health (KSA) (Reference No. A01731). Before participating in the study, all mothers were provided with a consent form explaining the study's purpose and their role. Participation was voluntary, and the mother could choose not to participate at any point during data collection. There were no physical or psychological risks for participants based on the nature of this study.

### 2.2 Sample size calculation and sampling technique

Convenience sampling was used to recruit participants from those attending the well-baby clinic. Details about the sample size are given in the standard equation below. This is based on an assumption of a 50% prevalence of good practices of CF according to the Food and Agriculture Organization (FAO) Guidelines (23). With a 95% confidence level and a confidence limit of 5%, the minimum sample size required is 385 participants.

$$n\geq \frac{z^{2}pq}{e^{2}}$$

$$n\geq \frac{1.96^{2}\times 0.50(1-0.50)}{0.05^{2}}=385$$

n: sample size.

z: z-score (standard normal deviate) corresponding to the 95% confidence level (1.96).

pq: variance of population (0.501).

e: allowable error (5%).

The four primary healthcare centers included in this study were selected using a two-stage sampling approach. In the first stage, Jeddah city was divided into its four broad geographical zones (north, south, east, and west) to ensure adequate geographic coverage of the population (24). Within each zone, one primary healthcare center was purposively selected based on three criteria: (1) the availability of a well-baby clinic offering both vaccination and healthy-child growth-monitoring services, since these services define the target population of mothers who regularly attend for their children's routine follow-up visits; (2) the center's location within the largest and most populous neighborhood in its respective zone, to maximize the potential participant pool and improve representativeness of the surrounding population; and (3) the accessibility and logistical feasibility of conducting data collection at the site during the study period. No random sampling procedure was used to select the healthcare centers. In the second stage, mothers attending the well-baby clinics at each of the four selected centers were recruited via convenience sampling; all eligible mothers who presented during the data-collection period were invited to participate. Mothers and their children were recruited, with approximately equal subsamples of 97 mother–child pairs enrolled from each of the four participating centers. Enrollment continued at each site until the target subsample size was reached.

### 2.3 Data collection

Data were collected using a structured questionnaire and anthropometric measurements of the children. Information was obtained through face-to-face interviews, and the questionnaires were completed by a trained researcher, as follows:

1. **General information:** It was collected for both mothers and children. Maternal data included nationality, age (in years), body mass index (BMI), marital status, highest level of education attained, employment status, monthly household income, and primary source of knowledge about complementary feeding. Child-related data covered age (in months), sex, birth weight, mode of delivery, number of siblings, birth order within the family, breastfeeding and exclusive breastfeeding status, and timing of breastfeeding initiation.
2. **Mother's KAP:** This section was based on the FAO validated instrument (23). It was used in similar research studies in different countries (14, 25–27). This instrument is composed of three domains:
  - Knowledge: It was assessed by seven questions that explore key aspects of infant and young child feeding, such as the recommended duration of breastfeeding, the appropriate age for introducing complementary foods, reasons for timely introduction, the choice and preparation of nutrient-rich porridges, food enrichment strategies, and methods to encourage children to eat. Each correct response scored 1, while incorrect answers scored 0. The total correct score was calculated for each mother.
  - Attitudes: Seven items assessed mothers’ attitudes regarding recommended feeding practices. These included confidence in food preparation, perceived importance and difficulty of providing dietary diversity, meal frequency, and continued breastfeeding beyond six months. Two types of scales were used based on the item: (1 = not difficult, 2 = neutral, 3 = difficult) or (1 = not good, 2 = not sure, 3 = good). The total score obtained for attitudes was calculated for each mother.
  - Practices: This domain assessed actual child feeding behaviors, including continued breastfeeding, minimum meal frequency, and dietary diversity. Minimum meal frequency was evaluated according to WHO/UNICEF recommendations based on age and breastfeeding status: breastfed infants aged 6–8 months should receive 2–3 complementary meals per day; breastfed children aged 9–23 months should receive 3–4 complementary meals plus 1–2 nutritious snacks; and non-breastfed children aged 6–23 months should receive at least 4 meals per day. Dietary diversity was measured using maternal recall of foods consumed by the child in the previous 24 h, classified into seven groups (grains, legumes/nuts, dairy products, flesh foods, eggs, vitamin A–rich fruits/vegetables, and other fruits/vegetables). A score of 1 was given for each food group consumed, and children were considered to meet minimum dietary diversity if they had consumed foods from at least four of the seven groups (12).
  - While the FAO KAP instrument has been applied in several countries (14, 25–27), its psychometric properties have also been specifically tested in an Arab population by Mohamed Ahmed Ayed, et al. (25). In their study on mothers of children aged 6–23 months in Egypt, these authors reported strong content validity (Content Validity Index = 0.96) and internal consistency (Cronbach's α = 0.88) for this same instrument (25). Given the linguistic, cultural, and regional similarities between Arab populations, this provides supportive, though not population-specific, evidence for the instrument's validity and reliability in an Arabic-speaking context. To further verify its clarity and comprehensibility in the Saudi Arabian setting, this study additionally conducted its own pilot testing before data collection. No major issues were identified, and no substantive modifications to the instrument were required before full data collection.
3. **Anthropometric Measurements:** Weight and length/height of children included in the study were measured by a trained nurse. Length or height was measured to the nearest 0.1 cm using an infant length board (Seca 233, Seca GmbH, Hamburg, Germany) for infants and toddlers unable to stand without support, and a portable height gauge (Seca 284, Seca GmbH, Hamburg, Germany) for all other children. Weight was measured to the nearest 0.1 kg using a scale with non-detachable weights, integrated into the same instruments (Seca 233 for infants and Seca 284 for older children); infants were weighed without clothes or with pre-weighed diapers, and older children were weighed in light clothing. The children's weight at birth and current age were verified using a birth certificate or the mother and child health booklet. The length and weight were converted to nutritional outcome indicators using the percentile system. The children's nutritional status was assessed using Z-scores in relation to the Saudi Ministry of Health Child Growth (28). The weight-for-length (WFL) index was classified as: wasted (<– 2 Z-score), normal (≥ −2 to ≤  + 1 Z-score), possible risk of overweight (> + 1 to ≤  + 2 Z-score), overweight (> + 2 to ≤  + 3 Z-score), and obese (> + 3 Z-score). The length-for-age (LFA) index was categorized as: severe stunting (<–3 Z-score), stunting (≥ −3 to < – 2 Z-score), normal (> −2 to ≤+3 Z-score), and tall (> + 3 Z-score). The weight-for-age (WFA) index was defined as: severely underweight (<–3 Z-score), underweight (≥ −3 to < – 2 Z-score), normal (> −2 to ≤+1 Z-score), overweight (> + 1 Z-score), and obese (> + 2 Z-score) (27). Consistent with WHO guidance, WFA was used exclusively to identify underweight and severe underweight, as this index does not account for a child's length and is not a valid tool for classifying overweight or obesity (11). Overweight and obesity were classified using weight-for-length (WFL) Z-scores, in line with WHO recommendations for children under 24 months of age.

### 2.4 Statistical analysis

All data analyses were conducted using R software, version 4.4.0 (R Foundation for Statistical Computing, Vienna, Austria). Data were presented using descriptive statistics in the form of frequencies and percentages for qualitative variables and means and SDs for quantitative variables. Pearson's chi-squared test was performed to assess the association between the studied parameters. The Spearman's correlation coefficient was applied to measure the strength and direction of the association between variables.

Multiple linear regression analyses were conducted to examine the associations between mothers' childcare knowledge, attitudes, practices, their sociodemographic characteristics, and children's anthropometric indices**.** Variables with a *p*-value <0.20 in the univariable analyses were considered for inclusion in the multivariable models. Continuous variables, including maternal age, maternal BMI, and family income, were entered as continuous predictors. Categorical variables, including educational level, residence, and working status, were treated as nominal variables and dummy-coded using (k−1) indicator variables, with primary education used as the reference category for educational level and not working used as the reference category for working status. The regression coefficients (*β*), standard errors (SE), *t*-statistics, *p*-values, and variance inflation factors (VIFs) were reported. Statistical significance was set at *p* < 0.05.

## 3 Results

### 3.1 General characteristics

A total of 390 mother–child pairs were enrolled in the study, exceeding the minimum required sample size of 385. The general characteristics of the mothers who participated in the study are summarized in Table 1. Most were Saudi nationals (70.0%), and more than half were between the ages of 30 and 39 (50.8%). Many mothers were either overweight (41.0%) or obese (19.2%). Most of the mothers were married (99.5%), and more than half had a bachelor's degree (52.3%), while only 21.0% were employed. Household income varied; 36.2% reported a monthly income of 5,000 to 9,999 SAR, and 21.8% earned less than 5,000 SAR. For sources of information on complementary feeding, digital media (74.4%) and relatives (73.6%) were the most mentioned, followed by books (29.0%) and health professionals (17.7%).

**Table 1:** General characteristics of the mothers of children aged 6–23 months who participated in the study, jeddah (*n* = 390).

Parameter	n	%
Nationality		
Saudi	273	70.0
Non-Saudi	117	30.0
Age (years)		
18–29	164	42.0
30–39	198	50.8
40–49	28	7.2
BMI (Kg/m^2^)		
<18.5	9	2.3
18.5–24.9	146	37.4
25–29.9	160	41.1
≥30	75	19.2
Marital status		
Married	388	99.5
Widowed/Separate/Divorce	2	0.5
Education level		
Primary education	21	5.4
High school	125	32.0
Diploma	21	5.4
Bachelor	204	52.3
Postgraduate	19	4.9
Employment		
Student	28	7.2
Working	82	21.0
Not working	280	71.8
Monthly household income (SAR)		
Less than 5,000 riyals	85	21.8
5,000 to < 10,000	141	36.2
10,000 to < 15,000	104	26.7
15,000 to < 20,000	45	11.5
≥20,000	15	3.8
Source of knowledge about complementary food^a^		
Health professional	69	17.7
Digital media	290	74.4
Relatives	287	73.6
Books	113	28.9
Other	3	0.8

BMI, Body Mass Index; SAR, Saudi Riyals.

a Source of knowledge about complementary food was a multiple-response item (mothers could select more than one source); percentages are calculated as the proportion of mothers selecting each option (denominato*r* = 390) and therefore sum to more than 100%.

The characteristics of the children who participated in the study are presented in Table 2 (*n* = 390). Nearly half of the children were aged 12 to 23 months (47.2%), with a balanced sex distribution (51.8% male, 48.2% female). Most had a birth weight of 2.5 kg or more (84.6%) and were delivered vaginally (59.0%). More than a quarter were firstborn (28.5%), while 27.9% had no siblings. The majority were breastfed at the time of the survey (87.4%). Among breastfed children (*n* = 341), only 31.7% were exclusively breastfed, and breastfeeding most often started within 2 h after birth (56.3%). Anthropometric indicators indicate that most children had a normal weight-for-age (76.9%), length-for-age (93.3%), and weight-for-length (71.8%). However, 22.8% were overweight, 2.6% were stunted, and 1.8% were obese, while severe undernutrition (severe underweight, severe stunting, or wasting) was very low (1.0% or less).

**Table 2:** General characteristics of the children aged 6–23 months who participated in the study (*n* = 390).

Parameter	n	%
Age (months)		
6–8	126	32.3
9–11	80	20.5
12–23	184	47.2
Sex		
Male	202	51.8
Female	188	48.2
Birth weight (Kg)		
<2.5 kg	60	15.4
≥2.5 Kg	330	84.6
Delivery mode		
Normal	230	59.0
Caesarean	160	41.0
Number of siblings		
0	109	27.9
1	102	26.2
2	90	23.1
3	57	14.6
4	24	6.2
≥5	8	2.1
Birth order of the child		
1	111	28.5
2	103	26.4
3	89	22.8
4	56	14.4
≥5	31	7.9
Baby breastfed		
Yes	341	87.4
No	49	12.6
Baby exclusively breastfed (*n* = 341)		
Yes	108	31.7
No	233	68.3
Initiation of breastfeeding (*n* = 341)		
Within 30 min after birth	49	14.4
Within 1 h after birth	85	24.9
Within 2 h 10	192	56.3
Others	15	4.4
Anthropometric Indicators		
*WFA*		
Severely underweight	1	0.3
Underweight	0	0.0
Normal	300	76.9
Overweight	89	22.8
*LFA*		
Severe stunting	4	1.0
Stunting	10	2.6
Normal	364	93.3
Tall	12	3.1
*WFL*		
Wasting	2	0.5
Normal	280	71.8
Risk of overweight	75	19.2
Overweight	26	6.7
Obese	7	1.8

All percentages are calculated based on the total sample (*n* = 390), except for baby exclusively breasted and breastfeeding initiation, for which percentages are calculated among breastfed children (*n* = 341).

WFA, Weight-for-Age; LFA, Length-for-Age; WFL, Weight-for-Length.

### 3.2 Indicators of complementary feeding

Minimum dietary diversity among infants and children aged 6 to 23 months (*n* = 390), broken down by breastfeeding status, is summarized in Table 3. It shows that 321 (82.3%) of the 390 children met the minimum dietary diversity criterion, while 69 (17.7%) did not. Among breastfed children (*n* = 341), dietary diversity increased significantly with age (*p* = 0.001), from 66.4% among children aged 6–8 months to 89.0% among those aged 9–11 months and 93.8% among those aged 12–23 months. Among non-breastfed children (*n* = 49), the proportion meeting minimum dietary diversity also varied significantly by age (*p* = 0.029), increasing from 47.4% at 6–8 months to 85.7% at 9–11 months and 82.6% at 12–23 months. Overall, the findings indicate that minimum dietary diversity was more frequently achieved among older children in both breastfeeding groups.

**Table 3:** Minimum dietary diversity among infants and children aged 6–23 months, by breastfeeding status and age group (*n* = 390).

Age (months)	Minimum dietary diversity		*P*-value
	Met n (%)	Not met n (%)	
Total	321 (82.3)	69 (17.7)	
Breastfed children (*n* = 341)	287 (84.2)	54 (15.8)	0.001
6–8 (*n* = 107)	71 (66.4)	36 (33.6)	
9–11 (*n* = 73)	65 (89.0)	8 (11.0)	
12–23 (*n* = 161)	151 (93.8)	10 (6.2)	
Non-breastfed (*n* = 49)	34 (69.4)	15 (30.6)	0.029
6–8 (*n* = 19)	9 (47.4)	10 (52.6)	
9–11 (*n* = 7)	6 (85.7)	1 (14.3)	
12–23 (*n* = 23)	19 (82.6)	4 (17.4)	

*P*-value calculated using the Chi-square test.

The distribution of children based on their age and adherence to the recommended nutrition, meeting the minimum meal frequency guidelines, is presented in Figure 1. Overall, a little more than 50% of the children met the minimum meal frequency guidelines, about 43% did not meet them, and only 5% exceeded them. For the 6–8 months age group, the highest percentage was recorded in the “recommended” category, at 15.90%. Similarly, for the 12–23 months age group, 26.92% fell into the “recommended” category. In contrast, among the 9–11 months age group, 10% of children were classified as having “less than recommended” meal frequency, marking the highest proportion of this category within that age group.

**Figure 1:**
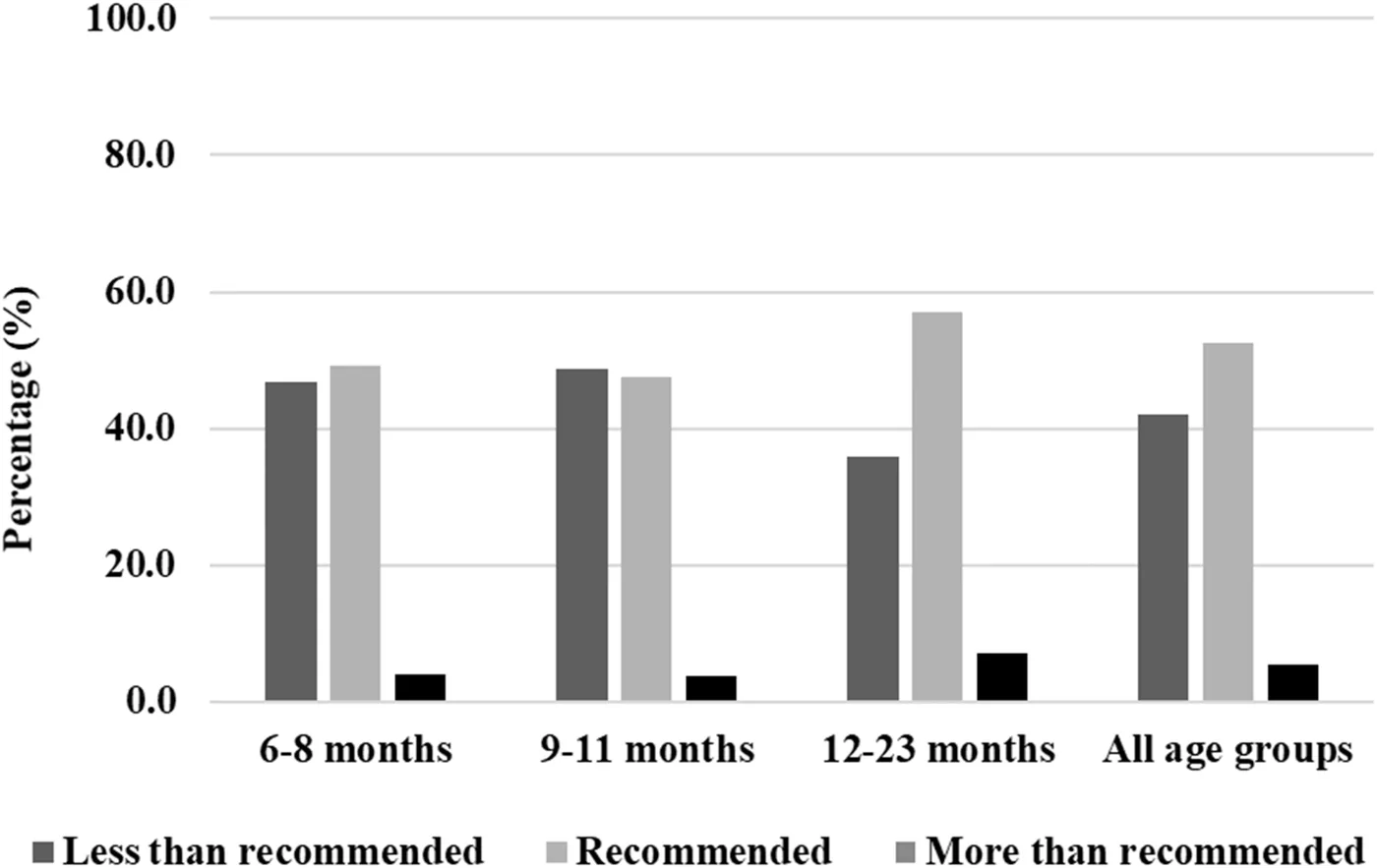
Frequency distribution of children aged 6–23 months according to whether the minimum recommended meal frequency was met, less than met, or exceeded, and by age group (*n* = 390).

### 3.3 Relationship between mothers' KAP and anthropometric indices of children

The KAP scores showed generally favorable results. The mean knowledge score was 4.62 ± 1.40 (median: 5.0) on a 0–7 scale. The attitude score was comparatively high, with a mean of 18.64 ± 2.46 (median: 19.0) on a 7–21 scale. The mean dietary diversity practice score was 3.86 ± 1.17 (median: 4.0) on a 0–7 scale (Table 4).

**Table 4:** Descriptive statistics (possible range, mea*n* ± SD, median) of maternal nutritional knowledge, attitude, and dietary-diversity practice scores (*n* = 390).

KAP Domain	Possible range	Mean ± SD	Median
Knowledge score	0–7	4.62 ± 1.40	5.0
Attitude score	7–21	18.64 ± 2.46	19.0
Practice (dietary diversity) score	0–7	3.86 ± 1.17	4.0

The relationship between mothers' nutritional knowledge, attitudes, and complementary feeding practices, as well as their children's anthropometric indices, is detailed in Table 5. A significant correlation was found between mothers’ knowledge and the three anthropometric indices: WFA, LFA, and WFL, with a *p*-value less than 0.05. Notably, WFA was the only index that demonstrated a significant correlation with all three maternal parameters—knowledge, attitude, and practice. However, it is important to note that while these relationships are statistically significant, they are relatively weak, as indicated by the R values ranging between 0.08 and 0.18.

**Table 5:** Spearman correlation coefficients between maternal nutritional knowledge, attitude, and complementary feeding practice scores and children's weight-for-age, length-for-age, and weight-for-length Z-score indices (*n* = 390).

Anthropometric indices	Knowledge		Attitude		Practice	
	r	P	r	P	r	P
WFA	0.18	<0.001	0.11	0.031	0.11	0.028
LFA	0.11	0.031	0.09	0.077	0.08	0.138
WFL	0.11	0.033	0.08	0.132	0.09	0.084

WFA, Weight-for-Age; LFA, Length-for-Age; WFL, Weight-for-Length; r, Spearman correlation coefficient; P, *p*-value.

The results of a multiple linear regression assessing the relationship between maternal sociodemographic characteristics, maternal BMI, nutritional knowledge, attitudes, and achieving the minimum meal diversity, along with children's growth measurements (*n* = 390), are presented in Table 6. For WFA, both maternal nutritional knowledge (*β* = 0.041, *p* = 0.013) and BMI (*β* = 0.016, *p* < 0.001) showed a significant association. For LFA, maternal working status (vs. not working) had a negative association (*β* = –0.058, *p* = 0.046). In contrast, nutritional knowledge (*β* = 0.026, *p* = 0.048) showed a positive association. No significant associations were observed for WFL.

**Table 6:** Multiple linear regression coefficients for associations between maternal sociodemographic characteristics, nutritional knowledge, attitude, and meeting minimum dietary diversity, and children's weight-for-age, weight-for-length, and length-for-age Z-score indices (n = 390).

Sociodemographic characteristics	*β*	SE	VIF	t	*p*-value
WFA					
Age (years)	0.002	0.003	1.179	0.721	0.471
Maternal BMI (kg/m^2^)	0.016	0.005	1.068	3.397	<0.001
Educational level					
High school vs. Primary	0.184	0.102	5.007	1.803	0.072
Diploma vs. Primary	0.161	0.138	2.134	1.174	0.241
Bachelor vs. Primary	0.164	0.104	5.971	1.575	0.116
Postgraduate vs. Primary	0.081	0.147	2.223	0.547	0.585
Working status					
Student vs. Not working	−0.104	0.087	1.125	−1.189	0.235
Working vs. Not working	0.012	0.062	1.408	0.186	0.852
Family income	0.018	0.024	1.385	0.766	0.445
Nutrition knowledge	0.041	0.016	1.135	2.508	0.013
Attitude towards nutrition	0.004	0.009	1.133	0.471	0.638
Meeting the minimum meal diversity	−0.004	0.051	1.135	−0.071	0.944
WFL					
Age (years)	−0.002	0.004	1.179	−0.387	0.699
Maternal BMI (kg/m^2^)	0.010	0.008	1.068	1.250	0.212
Educational level					
High school vs. Primary	0.228	0.168	5.007	1.361	0.174
Diploma vs. Primary	0.457	0.226	2.134	2.018	0.054
Bachelor vs. Primary	0.140	0.171	5.971	0.819	0.413
Postgraduate vs. Primary	0.470	0.242	2.223	1.939	0.053
Working status					
Student vs. Not working	−0.014	0.144	1.125	−0.095	0.924
Working vs. Not working	0.023	0.102	1.408	0.227	0.821
Family income	0.010	0.039	1.385	0.263	0.793
Nutrition knowledge	0.043	0.027	1.135	1.611	0.108
Attitude towards nutrition	0.009	0.015	1.133	0.564	0.573
Meeting the minimum meal diversity	−0.000	0.084	1.135	−0.003	0.997
LFA					
Age (years)	0.002	0.002	1.179	1.116	0.265
Maternal BMI (kg/m^2^)	0.004	0.004	1.068	1.012	0.312
Educational level					
High school vs. Primary	0.069	0.084	5.007	0.823	0.411
Diploma vs. Primary	0.194	0.113	2.134	1.723	0.086
Bachelor vs. Primary	0.055	0.085	5.971	0.646	0.519
Postgraduate vs. Primary	−0.045	0.121	2.223	−0.373	0.709
Working status					
Student vs. Not working	−0.085	0.072	1.125	−1.181	0.238
Working vs. Not working	−0.094	0.051	1.408	−1.847	0.046
Family income	0.031	0.019	1.385	1.611	0.108
Nutrition knowledge	0.026	0.013	1.135	1.981	0.048
Attitude towards nutrition	0.010	0.008	1.133	1.283	0.200
Meeting the minimum meal diversity	−0.034	0.042	1.135	−0.810	0.418

Primary education was used as the reference category for educational level, and “not working” was used as the reference category for working status.

BMI, Body Mass Index, WFA, Weight-for-Age; WFL, Weight-for-Length; LFA, Length-for-Age; β, unstandardized regression coefficient; SE, Standard Error; VIF, Variance Inflation Factor; t, t-statistic.

## 4 Discussion

This study provides valuable insights into the KAP of mothers regarding CF in Jeddah, Saudi Arabia. It also examines the relationship between these factors and the nutritional status of children aged 6 to 23 months. A key finding is that digital media serves as the main source of CF information for mothers, with 74.4% relying on it much more than on health professionals (17.7%). This trend is notable, especially given that more than half of the mothers in the sample (52.3%) have a bachelor's degree. In fact, the increasing reliance on social networks for health and nutrition information reinforces the evidence of how widespread and accessible these platforms are. However, the lack of regulation often leads to misinformation, which could pose serious risks to public health. This concern is highlighted in research by Segado-Fernández et al. (2025) (29), Viviani et al. (2027) (30), and Alduraywish et al. (2020) (31). Trusting non-clinical sources such as digital media can lead to advice that does not match evidence-based recommendations, including those from the World Health Organization (1, 2, 7). Therefore, it is crucial to raise awareness about the need for reliable and evidence-based health information sources. This is particularly important for mothers of young children. The age group in this study falls within the first 1,000 days of life, as a vital period for optimal growth, cognitive development, and long-term health (32).

The anthropometric data indicate that the majority of the children in this study fall within the normal range according to WFA, LFA, and WFL. It is important to note that the overweight prevalence estimated by WFA (22.8%) was substantially higher than that estimated by WFL (6.7%). This divergence is expected since WHO guidance specifies that WFA reflects only whether a child's weight is appropriate for their chronological age and cannot distinguish a child who is heavy because they are long/tall from a child who is heavy relative to their body length (11). Given that 19.2% of children in this sample were classified as tall-for-age, it is likely that a substantial proportion of children flagged as ‘overweight’ by WFA were in fact of normal weight relative to their length. We therefore consider the WFL-based estimate to be the more valid indicator of overweight prevalence in this cohort, and interpret the WFA finding as reflecting the combined influence of linear growth and weight gain rather than excess adiposity *per se*. Accordingly, 6.7% of children in this study are classified as overweight according to WFL, a figure that mirrors national data showing a rise in childhood overweight (33) and aligns with global concerns about the increasing prevalence of childhood obesity (34). This evidence further underscores the need for CF education to address not only dietary adequacy but also the prevention of overnutrition and the promotion of healthy food choices.

The analysis of CF practices revealed generally positive outcomes for dietary diversity, particularly among older children. The proportion of children meeting the minimum dietary diversity in the total study population was 82.3%. Among breastfed children, the proportion meeting minimum dietary diversity increased steadily with age, from 66.4% at 6–8 months to 89.0% at 9–11 months and 93.8% at 12–23 months. Among non-breastfed children, dietary diversity rose sharply between 6 and 8 months (47.4%) and 9–11 months (85.7%), and remained comparably high at 12–23 months (82.6%). Similar trends were reported by Ahmed & Salih in 2019 (17). The age-related improvement may reflect progressive exposure to a wider variety of foods as infants grow older (35); however, non-breastfed children showed consistently lower dietary diversity than breastfed children within the youngest age group in particular, and the gap during the early CF period (6–8 months) is particularly concerning given its critical role in shaping long-term feeding habits and preventing nutrient deficiencies (36). In addition, meal frequency emerged as an area requiring attention, with a notable proportion of children, especially in the 9–11 months age group, receiving less than the recommended number of meals. This is consistent with previous Saudi studies' findings that nearly 42% of children did not receive the minimum number of meals daily (17), suggesting a persistent gap in understanding the importance of frequent feeding to meet the high energy needs of infants (2).

The primary objective of this study was to investigate the association between maternal KAP and child nutritional status. Maternal nutritional knowledge showed significant, although weak, positive correlations with WFA, LFA, and WFL. In the multivariable analysis, maternal nutritional knowledge remained positively associated with WFA and LFA. This aligns with the global consensus that maternal knowledge is a key determinant of improved child feeding practices and nutritional outcomes (37–39). Maternal BMI was also independently and positively associated with WFA, whereas no significant association was observed between maternal BMI and WFL or LFA. The association between maternal BMI and WFA should be interpreted cautiously because WFA reflects body weight relative to age and does not account for attained length. Therefore, this finding should not be interpreted as evidence that maternal BMI was associated with excess weight-for-length or overweight in the children. The relatively small coefficients observed in the regression models also indicate that these maternal characteristics explain only a limited proportion of the variation in child anthropometric status. Additionally, KAP were all significantly and positively correlated with WFA, reflecting the influence of maternal KAP on acute nutritional status. These findings are consistent with previous studies (24, 40). Interestingly, the regression analysis in this study also revealed that a mother's working status was negatively associated with LFA. This index captures longer-term cumulative effects on a child's growth. Taken together, these results highlight the potential influence of socioeconomic factors on food security and access to high-quality, diverse foods necessary for healthy development (41).

The positive association observed between maternal BMI and WFA in the present study is consistent with evidence suggesting that maternal adiposity is associated with offspring growth and early-life obesity risk. In a large register-based cohort study of 30,197 mother–child pairs, Österroos et al. (42) found that maternal BMI in early pregnancy was associated with subsequent childhood growth; children of mothers who were overweight or obese showed an increase in BMI z-scores from 18 months onward, and maternal obesity, particularly when accompanied by excessive gestational weight gain, was associated with a higher risk of overweight or obesity at 4 years. These findings suggest that maternal adiposity may remain relevant to child growth beyond infancy, although the exposure in that study was maternal BMI measured during early pregnancy rather than contemporaneous maternal BMI. More directly relevant to the age group examined in the present study, a recent case-control study of infants and young children below 2 years reported that maternal BMI >25 kg/m^2^ at enrolment was independently associated with childhood obesity (adjusted OR 11.25, 95% CI 3.66–34.49), while maternal pre-conceptional BMI >25 kg/m^2^ was also identified as a risk factor in univariate analyses (43). Importantly, that study used weight-for-length or BMI-for-age >+3 SD to define obesity and therefore assessed an outcome related to excess weight more specifically than WFA alone. Taken together, these findings provide supportive context for the positive association between maternal BMI and WFA observed in our study. However, the comparison should be interpreted cautiously because our study measured maternal BMI contemporaneously with the child's anthropometry and used WFA as the outcome, whereas the cited studies assessed early-pregnancy, pre-conceptional, or current maternal BMI in relation to overweight or obesity. Maternal BMI in our study may reflect a combination of familial, genetic, behavioral, and environmental factors, and the cross-sectional design precludes establishing temporality or causality. Therefore, our finding should be interpreted as an association with weight-for-age rather than evidence that maternal BMI causes excess weight or obesity in the child.

This study has some limitations that should be discussed. The cross-sectional design precludes the establishment of causal relationships between KAP and nutritional outcomes. In addition, the use of convenience sampling in well-baby clinics may limit the generalizability of the findings to the broader population of Jeddah or Saudi Arabia, as it may not capture mothers who do not regularly utilize these services. However, it is worth mentioning that the direct measurement of anthropometric data by trained personnel is a strength, as it is more reliable than parent-reported measures. Moreover, the use of a valid tool by FAO to assess the KAP of mothers related to CF is an additional strength, as it enhances the credibility of the findings and provides valuable insights into this research study. Finally, although maternal BMI was included in the multivariable analysis, BMI was measured contemporaneously with the child's anthropometric status rather than before pregnancy. Consequently, the temporal relationship between maternal BMI and child growth cannot be established, and the observed association should not be interpreted causally. Residual confounding by shared genetic, behavioral, and household environmental factors may also remain.

## 5 Conclusion

In conclusion, this study found generally favorable dietary diversity practices among older infants, although meal frequency remains an area requiring attention. According to WFL, 6.7% of children were overweight. Maternal nutritional knowledge was positively associated with WFA and LFA, while maternal BMI was independently and positively associated with WFA. Maternal working status and family income were also associated with LFA. The relatively small effect sizes indicate that these factors should be considered as part of a broader set of determinants of child growth rather than as independent explanations of nutritional status. Given the cross-sectional design, the observed associations should not be interpreted causally. Strengthening evidence-based complementary-feeding counseling through healthcare professionals, complemented by reliable digital nutrition information, may support appropriate feeding practices and healthy child growth. At the same time, official, evidence-based digital campaigns led by the Ministry of Health are necessary to combat misinformation and provide accessible, trustworthy resources. Together, these interventions, which support the goals of Saudi Vision 2030′s Health Sector Transformation Program, are vital to improving complementary feeding practices and securing the long-term health and development of children in Saudi Arabia.

## Data Availability

The raw data supporting the conclusions of this article will be made available by the authors, without undue reservation.

## References

[1] WHO. Infant Nutrition. Geneva: World Health Organization (2019). Available online at: https://www.who.int/health-topics/infant-nutrition

[2] WHO. Infant and Young child Feeding: Model chapter for Textbooks for Medical students and Allied health Professionals. Geneva: World Health Organization (2009). Available online at: https://iris.who.int/handle/10665/44117 23905206

[3] WHO. Indicators for Assessing infant and Young child Feeding practices: Part 1 Definition. Geneva: World Health Organization (2008). Available online at: https://www.who.int/publications/i/item/9789241596664

[4] Pietrobelli A Agosti M, MeNu Group. Nutrition in the first 1,000 days: ten practices to minimize obesity emerging from published science. Int J Environ Res Public Health. (2017) 14(12):1491. 10.3390/ijerph14121491 29194402 PMC5750909

[5] UNICEF. Maternal Nutrition programming Guidance. New York: United Nations Children’s Fund (2021). Available online at: https://www.unicef.org/media/115361/file/Maternal%20Nutrition%20Programming%20Guidance.pdf

[6] Mameli C Mazzantini S Zuccotti GV. Nutrition in the first 1000 days: the origin of childhood obesity. Int J Environ Res Public Health. (2016) 13(9):838. 10.3390/ijerph13090838 27563917 PMC5036671

[7] WHO. Infant and Young child Feeding. Geneva: World Health Organization (2023). Available online at: https://www.who.int/news-room/fact-sheets/detail/infant-and-young-child-feeding

[8] Zhang Y Huang X Yang Y Liu X Yang C Wang A. Double burden of malnutrition among children under 5 in poor areas of China. PLoS One. (2018) 13(9):e0204142. 10.1371/journal.pone.0204142 30222775 PMC6141094

[9] WHO. Complementary Feeding. Geneva: World Health Organization (2019). Available from: Available online at: https://www.who.int/health-topics/complementary-feeding

[10] WHO. Accelerating Nutrition improvements (ANI): Mapping of Stakeholders and Nutrition actions in Three scaling-up Countries in sub-Saharan Africa: Report of a Meeting, 27–28 February 2014, Addis Ababa, Ethiopia. Geneva: World Health Organization (2014).

[11] WHO. Guideline: Assessing and Managing children at Primary health-care Facilities to Prevent overweight and Obesity in the Context of the Double burden of Malnutrition. Geneva: World Health Organization (2017). Available online at: https://www.who.int/publications/i/item/9789241550123 29578661

[12] WHO. Indicators for Assessing infant and Young child Feeding practices: Definitions and Measurement methods. Geneva: World Health Organization (2021). Available online at: https://www.who.int/publications/i/item/9789240018389

[13] Edith M Priya L. Knowledge, attitude, and practice (KAP) survey on dietary practices in prevention of malnutrition among mothers of under-five children. Manipal J Nurs Health Sci. (2016) 2(2):19–24.

[14] Forh G Apprey C Frimpomaa Agyapong NA. Nutritional knowledge and practices of mothers/caregivers and its impact on the nutritional status of children 6–59 months in sefwi wiawso municipality, western-north region, Ghana. Heliyon. (2022) 8(12):e12330. 10.1016/j.heliyon.2022.e12330 36590498 PMC9798164

[15] WHO. Global Strategy for Infant and Young child Feeding. Geneva: World Health Organization (2003). Available online at: https://www.who.int/publications/i/item/9241562218

[16] Global Nutrition Report. Country Nutrition profiles. Bristol: Development Initiatives (2022). Available online at: https://globalnutritionreport.org/resources/nutrition-profiles/asia/western-asia/saudi-arabia/

[17] Ahmed A Salih O. Infant and young child complementary feeding in Saudi arabia: timely introduction, frequency and diversity. Pak J Nutr. (2019) 18:216–25. 10.3923/pjn.2019.216.225

[18] Al-Mutairi WH Sulaiman AA. Knowledge, attitude, and practices regarding complementary feeding among mothers of children aged 6–24 months in primary healthcare centers in unaizah city, Saudi arabia. Int J Med Dev Ctries. (2021) 5(5):1134. 10.24911/IJMDC.51-1613562021

[19] Saaka M. Relationship between mothers’ Nutritional knowledge in childcare practices and the growth of children living in impoverished rural communities. J Health Popul Nutr. (2014) 32(2):237–48. 25076661 PMC4216960

[20] Angeles-Agdeppa I Monville-Oro E Gonsalves JF Capanzana MV. Integrated school based nutrition programme improved the knowledge of mother and schoolchildren. Matern Child Nutr. (2019) 15(Suppl 3):e12794. 10.1111/mcn.12794 31148401 PMC6593821

[21] Health Sector Transformation Program. Kingdom of Saudi arabia: national transformation program 2020. In: Saudi Vision 2030. Riyadh: Kingdom of Saudi Arabia 2021. Available online at: https://www.vision2030.gov.sa/en/explore/programs/health-sector-transformation-program

[22] Ghandour RM Jones JR Lebrun-Harris LA Minnaert J Blumberg SJ Fields J. The design and implementation of the 2016 national survey of children’s health. Matern Child Health J. (2018) 22(8):1093–102. 10.1007/s10995-018-2526-x 29744710 PMC6372340

[23] Food and Agriculture Organization of the United Nations. Guidelines for Assessing nutrition-related Knowledge, Attitudes and Practices. Rome: Food and Agriculture Organization of the United Nations (2014).

[24] Kattan W. The state of primary healthcare centers in Saudi arabia: a regional analysis for 2022. PLoS One. (2024) 19(9):e0301918. 10.1371/journal.pone.0301918 39250494 PMC11383242

[25] Mohamed Ahmed Ayed M Kamel Yousef Ali F Sayed Masoed Sayed E. Effect of mothers’ Nutritional knowledge, attitude, and practices in childcare on the growth of children. Egypt J Health Care. (2021) 12(2):371–82. 10.21608/ejhc.2021.161003

[26] Mutuku JN Ochola S Osero J. Maternal knowledge and complementary feeding practices and their relationship with nutritional status among children 6−23 months old in pastoral community of marsabit county, Kenya: a cross-sectional study. Curr Res Nutr Food Sci J. (2020) 8(3):862–76. 10.12944/CRNFSJ.8.3.17

[27] Bimpong KA Cheyuo EKE Abdul-Mumin A Ayanore MA Kubuga CK Mogre V. Mothers’ Knowledge and attitudes regarding child feeding recommendations, complementary feeding practices and determinants of adequate diet. BMC Nutr. (2020) 6(1):67. 10.1186/s40795-020-00393-0 33292706 PMC7706028

[28] El Mouzan MI Shaffi A Salloum AA Alqurashi MM Herbish AA Omer AA. Z-score growth reference data for Saudi preschool children. Ann Saudi Med. (2017) 37(1):10–5. 10.5144/0256-4947.2017.10 28151451 PMC6148975

[29] Segado-Fernández S Jiménez-Gómez B Jiménez-Hidalgo PJ Lozano-Estevan MC Herrera-Peco I. Disinformation about diet and nutrition on social networks: a review of the literature. Nutr Hosp. (2025) 42(2):366–75. 10.20960/nh.05533 40008658

[30] Viviani M Pasi G. Credibility in social media: opinions, news, and health information—a survey. Wiley Interdiscip Rev Data Min Knowl Discov. (2017) 7(5):e1209. 10.1002/widm.1209

[31] Alduraywish SA Altamimi LA Aldhuwayhi RA AlZamil LR Alzeghayer LY Alsaleh FS. Sources of health information and their impacts on medical knowledge perception among the Saudi Arabian population: cross-sectional study. J Med Internet Res. (2020) 22(3):e14414. 10.2196/14414 32191208 PMC7118549

[32] Aguayo VM Britto PR. The first and next 1000 days: a continuum for child development in early life. Lancet. (2024) 404(10467):2028–30. 10.1016/S0140-6736(24)02439-5 39571588

[33] AlEnazi S AlAjlan R AlKhalaf H Abolfotouh M Alharbi O Alfawaz R. Prevalence of obesity among children and adolescents in Saudi arabia: a multicenter population-based study. Saudi J Med Med Sci. (2023) 11(1):19–25. 10.4103/sjmms.sjmms_417_22 36909009 PMC9997853

[34] Zhang X Liu J Ni Y Yi C Fang Y Ning Q. Global prevalence of overweight and obesity in children and adolescents: a systematic review and meta-analysis. JAMA Pediatr. (2024) 178(8):800–13. 10.1001/jamapediatrics.2024.1576 38856986 PMC11165417

[35] Paroche M M Caton SJ Vereijken CM Weenen H Houston-Price C. How infants and young children learn about food: a systematic review. Front Psychol. (2017) 8:1046. 10.3389/fpsyg.2017.01046 28790935 PMC5524770

[36] D’Auria E Borsani B Pendezza E Bosetti A Paradiso L Zuccotti GV. Complementary feeding: pitfalls for health outcomes. Int J Environ Res Public Health. (2020) 17(21):7931. 10.3390/ijerph17217931 PMC7662522 33137971

[37] Abuya BA Ciera J Kimani-Murage E. Effect of mother’s education on child’s nutritional status in the slums of Nairobi. BMC Pediatr. (2012) 12:80. 10.1186/1471-2431-12-80 22721431 PMC3444953

[38] Fadare O Amare M Mavrotas G Akerele D Ogunniyi A. Mother’s nutrition-related knowledge and child nutrition outcomes: empirical evidence from Nigeria. PLoS One. (2019) 14(2):e0212775. 10.1371/journal.pone.0212775 30817794 PMC6394922

[39] Akeredolu IA Osisanya JO Seriki-Mosadolorun JS Okorafor U. Mothers’ Nutritional knowledge, infant feeding practices and nutritional status of children (0−24 months) in lagos state, Nigeria. Eur J Nutr Food Saf. (2014) 4(4):364–74. 10.9734/EJNFS/2014/7604

[40] Randhawa S Choudhury M Choudhary DG Ballala R Hegde S Barman P. Influence of mothers’ And frontline health workers’ Knowledge, attitude, and practices on infant and young child feeding and child nutrition: a cross-sectional study in aspirational districts of assam, India. Front Nutr. (2024) 11:1413867. 10.3389/fnut.2024.1413867 39777072 PMC11705560

[41] Shaker-Berbari L Qahoush Tyler V Akik C Jamaluddine Z Ghattas H. Predictors of complementary feeding practices among children aged 6–23 months in five countries in the Middle East and north Africa region. Matern Child Nutr. (2021) 17(4):e13223. 10.1111/mcn.13223 34137179 PMC8476411

[42] Österroos A Lindström L Wikman P Forslund A Wikström A-K Sundström Poromaa I. Maternal body mass index, gestational weight gain, and early childhood growth: a register-based cohort study. Acta Obstet Gynecol Scand. (2024) 103(11):2171–82. 10.1111/aogs.14961 PMC11502434 39231783

[43] Pushparajan RRM Sreenivasan P Sarasam AA. Risk factors of obesity among infants and young children below 2 years of age: a case control study. Indian J Med Res. (2026) 163(4):450–5. 10.25259/IJMR_2621_2025 42165734 PMC13224210

